# Leveraging Social Media and AI for Early Community Mental Health Support

**DOI:** 10.7759/cureus.95047

**Published:** 2025-10-21

**Authors:** Kaden Bunch, David Nguyen, Giovanni Kozel, Thanh V Doan, Emily Lin

**Affiliations:** 1 Medicine, The Warren Alpert School of Medicine, Brown University, Providence, USA; 2 Medicine, Michael G. DeGroote School of Medicine at McMaster University, Ontario, CAN; 3 Neurosurgery, The Warren Alpert School of Medicine, Brown University, Providence, USA; 4 Medicine, Johns Hopkins University School of Medicine, Baltimore, USA; 5 Internal Medicine, Brigham and Women's Hospital, Boston, USA

**Keywords:** artificial intelligence, community-based intervention, large language models, machine learning, mental health, public health care, public psychiatry, social media

## Abstract

Background

Mental health conditions have become a leading cause of disability worldwide, yet stigma, financial barriers, and limited access to care impede effective treatment. Amid these challenges, more people are turning to online platforms like Reddit to express psychological distress and seek informal support. However, these platforms often lack mechanisms to guide users toward professional care. This study explores a community-facing framework that leverages natural language processing and large language models (LLMs) to detect mental health concerns in social media posts and generate personalized support options.

Methods

We trained and evaluated multiple machine learning classifiers, including Logistic Regression, Random Forest, XGBoost, and DistilBERT, using the Reddit SuicideWatch and Mental Health Collection datasets for multilabel classification of mental health conditions, including depression, anxiety, bipolar disorder, and suicidal ideation. High-confidence predictions from these models were then used to prompt Llama 3.1 8B Turbo LLM to generate personalized mental health resources.

Results

Among the models, DistilBERT achieved the highest performance, with an area under the receiver operating characteristic curve of 0.916 (95% CI: 0.912-0.921), an F1 score of 0.762 (95% CI: 0.753-0.771), and an accuracy of 0.761 (95% CI: 0.752-0.770). Using these predictions, the LLM generated tailored resources matched to the identified mental health concerns.

Conclusion

By connecting symptom detection with resource generation, this framework aims to lower common barriers to mental healthcare, especially for individuals hesitant to seek traditional support. Instead of viewing classification as an endpoint, our approach shows how detection can lead to intervention. Linking symptom recognition with tailored resource creation, this work underscores AI’s potential to enable scalable, community-based mental health outreach that complements traditional care delivered by licensed mental health professionals in clinical settings.

## Introduction

The landscape of mental health

The incidence of mental health conditions is on the rise in the United States, increasing to 23.1% of U.S. adults in 2022 from 18.1% in 2009 [1]. In 2022, over 49,000 people died by suicide in the United States, equivalent to one death every 11 minutes [2], accounting for the highest number of deaths by suicide per year ever recorded in the United States [3]. Globally, mental health disorders are among the top 10 leading causes of loss of health, accounting for 17.2% of total years lived with disability in 2021. The global burden of mental disorders has especially been rising since 2020 in the wake of the COVID-19 pandemic [4]. 

Mental health conditions exhibit a high mortality rate, with an estimated 14.3% of deaths attributed to mental disorders per year globally [5]. In the United States, what have been termed “deaths of despair” (deaths from drugs, alcohol, and suicide) have more than doubled between the 1960s and 2017 and continue to rise [6]. Socioeconomic outcomes suffer across the lifespan, from lower school attendance and higher rates of school dropout to decreased employment and decreased earnings [6]. 

Mental health disorders are especially prevalent among vulnerable populations, including low-income individuals, racial and ethnic minorities, LGBTQ+ individuals, and those living in rural or underserved areas, for whom access to and availability of treatment can be particularly challenging [7,8]. The Substance Abuse and Mental Health Services Administration (SAMHSA) found that 29% of people suffering from serious mental illness did not know where to go for mental health services [6]. Stigma was also a significant barrier, as 12% reported concerns about confidentiality, and 11% were worried that other people would have a negative opinion [6]. Other barriers to mental healthcare include financial challenges, shortages in the mental health workforce, and uneven geographic distribution of mental health providers [8]. 

Prior evidence, project aims, and rationale

Early Community Mental Health Support and Digital Interventions

Early community-based mental health support has traditionally relied on local resources such as peer support groups, school- or workplace-based counseling, community clinics, and outreach programs aimed at identifying and assisting individuals at risk. These programs, when utilized, are excellent at lowering barriers to care, offering timely intervention, and providing psychosocial support within a familiar local environment. Vulnerable populations, however, facing stigma, limited access, and lack of funds, have prompted the growth of nontraditional services such as mobile apps and online support groups [8,9].

Social Media as a Platform for Mental Health Engagement

Widespread social media use has facilitated the study of depressive and anxiety disorders through online platforms such as Reddit [10]. Social media can help facilitate candid and expressive self-disclosure of mental disorder symptoms [11]. To date, most mental health studies involving Reddit have focused on classifying and predicting symptom patterns based on user-generated data, many of which utilize machine learning techniques for analysis [10]. Many such studies were focused on binary classification (e.g., depression or not depression), though a handful expanded the scope to include multilabel classification of mental disorders [11,12]. A handful of studies also suggested user-focused interventions, including embedding especially helpful posts into Reddit community guidelines [13], connecting people to other users facing similar struggles [14], and referring users to appropriate subreddits that could offer further support [15]. Although peer support can be immensely valuable, its use as a complement to formal interventions, along with robust collaborative efforts and support from professional resources, is important in facilitating a multimodal, more comprehensive approach to mental healthcare [16]. 

Overview of AI and Machine Learning Models

To support the analysis of social media text, this study leveraged several AI and machine learning methods. Natural language processing (NLP), a field of AI focused on understanding and generating human language, enabled the extraction of meaningful patterns from Reddit posts. Large language models (LLMs), such as Llama 3.1, build on NLP using deep learning to generate context-aware text and personalized recommendations [17]. BERT (Bidirectional Encoder Representations from Transformers) provides contextualized word representations [18], while DistilBERT offers a smaller, faster version with comparable performance [19]. For structured machine learning, we employed eXtreme gradient boosting (XGBoost) and Random Forest (RF), which combine decision trees for accurate prediction and classification, alongside Logistic Regression (LR), a widely used method for classifying categorical outcomes. Together, these models allowed us to identify mental health concerns in posts and generate tailored resources for users [20]. Unlike peer support alone, this approach enables a multimodal framework that integrates symptom detection with actionable guidance, bridging gaps between community engagement and professional care [16].

Study Aims

This study is characterized by several aims, as detailed below. 

We aim to develop a tool that can be used by individuals and healthcare providers to identify specific patterns of mental health symptoms based on individuals’ written expressions of their experiences. For this purpose, we will use the Reddit SuicideWatch and Mental Health Collection (SWMH) for Suicidal Ideation and Mental Disorder Detection database [21]. Specifically, our goal is to achieve multilabel classification of mental health symptom patterns, which can expand the scope and applicability of our model beyond the capabilities of binary classification. 

We furthermore aim to use this classification to direct users to personalized, professional, and peer-support mental health resources that target each individual patient’s needs. These tailored resources can help address an unmet need, given that prior interventions in this area have primarily focused solely on peer support. The grounding of LLM-generated recommendations via proven text classification models and user data can enhance the effectiveness of this approach.

We hope to utilize this tool to address some of the common barriers to mental healthcare detailed above, including a large proportion of people reporting that they did not know what resources they could access for their symptoms [6]. Furthermore, such a tool would come with the additional benefit of protecting confidentiality and decreasing the potential stigma that arises with candid discussion of mental health symptoms with other people, thus lowering the barrier to seeking treatment. With the known barriers in access to mental healthcare, this approach seeks to create an additional avenue of access to timely, individualized, life-saving care for patients.

Rationale for Symptom-Level Classification of Mental Health Conditions

Although depression, anxiety, bipolar disorder, and suicidal ideation often exhibit overlapping symptoms and may co-occur within the same individual, early identification of symptom patterns remains important before a formal diagnosis can be made. Multilabel classification allows the model to capture these comorbidities and overlapping presentations by assigning multiple labels to a single post, reflecting the complex nature of mental health while identifying more prominent themes [12]. Professional resources such as crisis lines or general counseling are constructed to serve multiple mental health conditions in a similar fashion. Within our model, while addressing the multiple labels adds value to resource recommendation, the addition of identifying the prominent theme in the user's social media post allows for the generation of more tailored, context-specific recommendations, including peer-support forums, and self-help interventions that align with the user’s expressed needs [14-16].

Preliminary symptom-level classification provides a structured framework for AI-assisted triage, facilitating timely, individualized support in non-clinical settings while maintaining confidentiality and scalability [10,11]. This approach not only enhances the relevance of the AI-generated guidance but also ensures that interventions can be prioritized based on the combination and severity of symptoms identified, complementing traditional pathways [16]. 

## Materials and methods

Dataset

**Figure 1:**
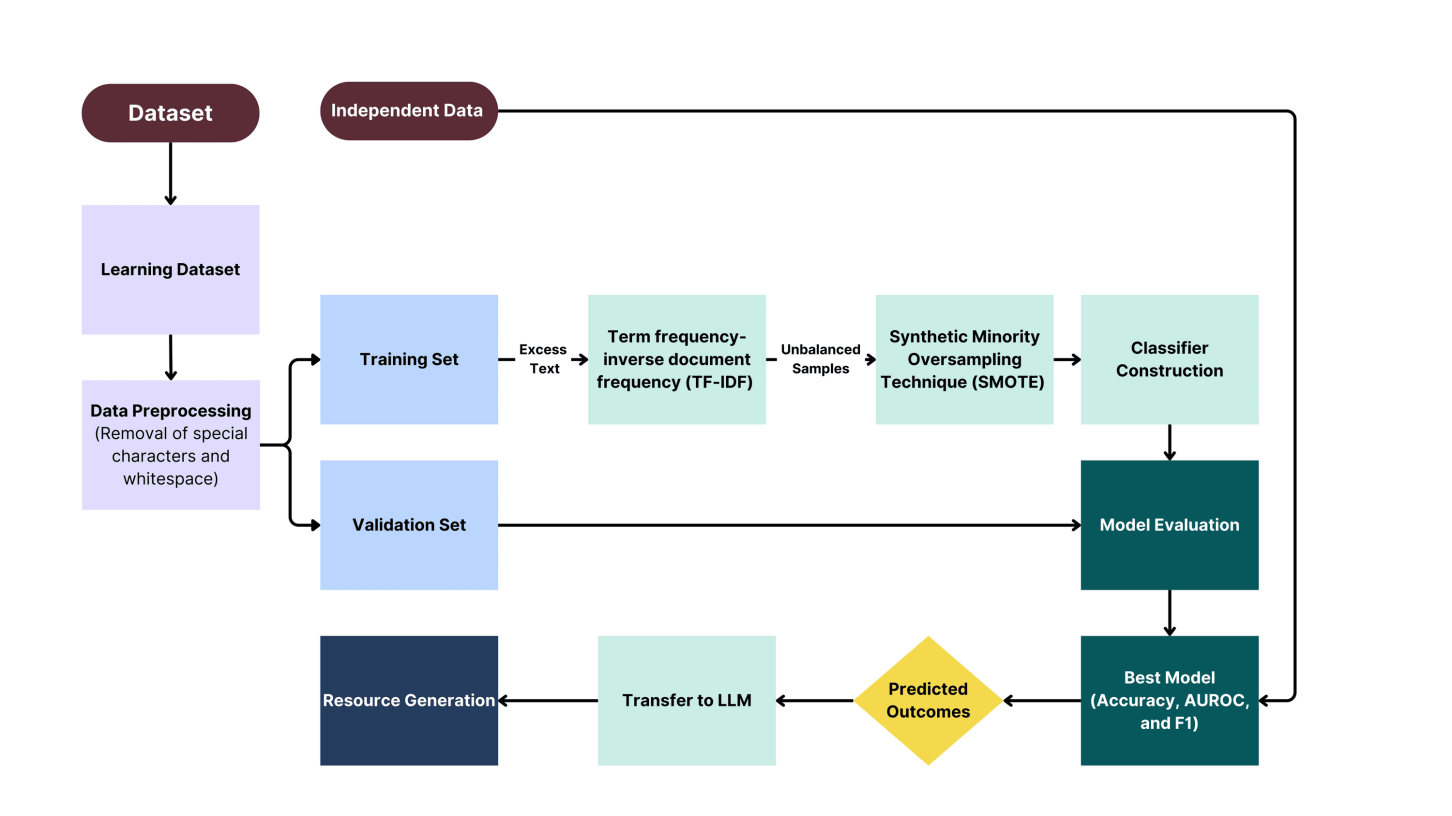
Flowchart of data processing and resource generation pipeline. This flowchart illustrates the key steps in analyzing social media data for mental health support. The process begins with dataset acquisition, followed by data preprocessing to prepare for analysis. The preprocessed data is then used to train machine learning models, which are evaluated for accuracy and reliability. Finally, the classified outcomes are passed to a large language model (LLM) to generate tailored mental health resources, such as counseling and crisis support, based on individual needs.

The study utilized the Reddit SuicideWatch and Mental Health Collection (SWMH) dataset [21]. Due to the nature of text data, a comprehensive methodology encompassing data preprocessing, feature extraction, model training, and evaluation was performed to identify the best machine learning model for LLM integration (Figure 1).

Data preprocessing

Initially, the datasets were loaded into separate training and testing data frames with an examination of their shape and label distribution to ensure data integrity and balance. Text cleaning was then performed on both datasets to remove special characters, excessive white space, and convert text to lowercase. Furthermore, text entries from the “offmychest” community were excluded from training and analysis to preserve predictive value for posts belonging to mental health-focused communities. 

**Figure 2:**
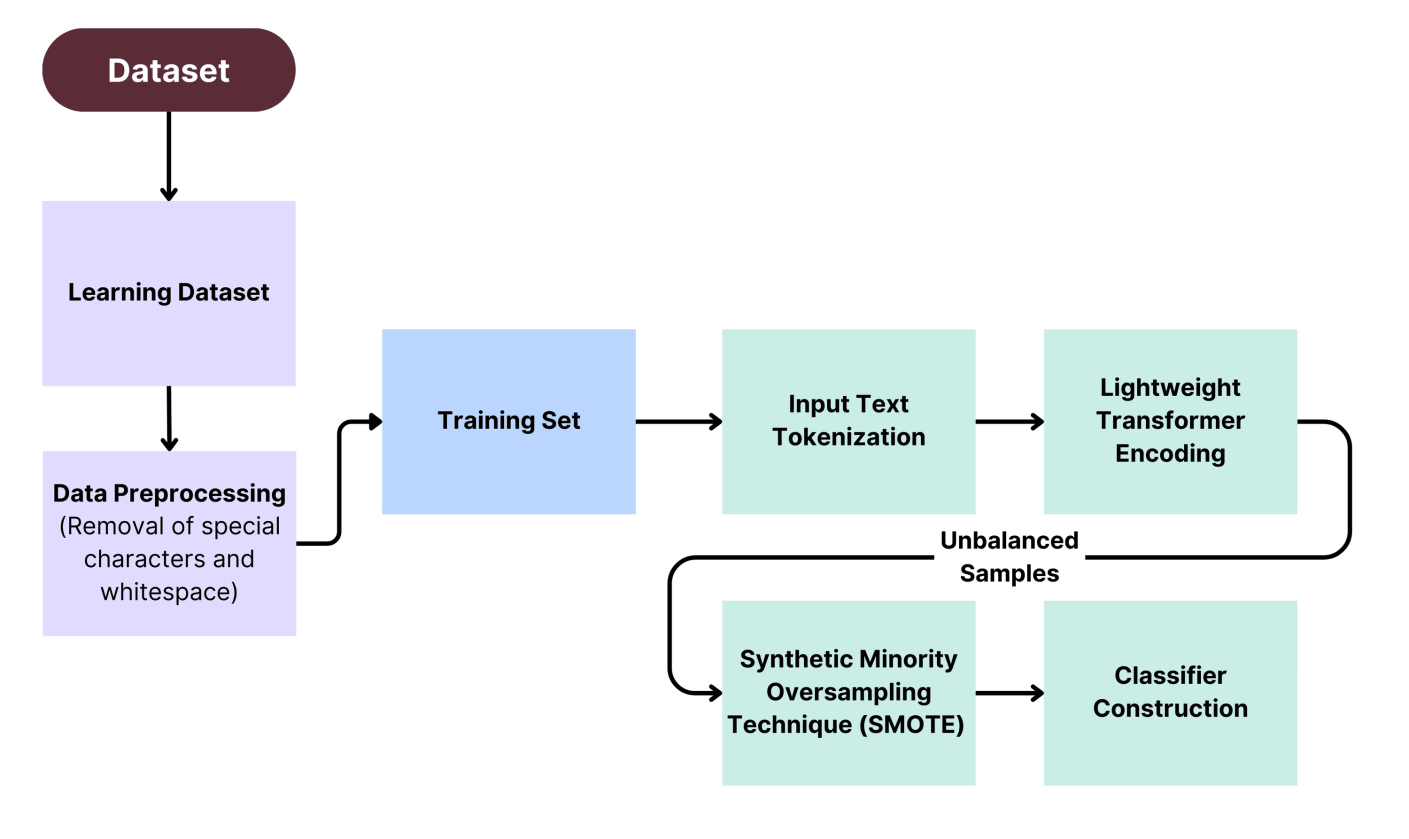
Flowchart of data pre-processing for the DistilBERT model. This flowchart illustrates the key steps, such as text tokenization, label encoding, oversampling mitigators, and consequent classifier construction.

For the DistilBERT model detailed in Figure 2, label encoding and text tokenization were carried out to align with DistilBERT’s input format and pre-trained vocabulary. Furthermore, dynamic padding and batch preparation were leveraged, optimizing memory efficiency while allowing padding tokens to be ignored using attention masks [19]. Lastly, a validation split was created from the training data to periodically evaluate the model’s performance on unseen data, helping to identify overfitting and preventing overreliance on patterns that may have been present in the dataset.

Feature extraction

Feature extraction was then performed. This involved converting the text data into numerical features using Term Frequency-Inverse Document Frequency (TF-IDF) vectorization. This technique converts text data into a matrix of TF-IDF features, allowing the text to be put in machine learning models [22]. 

Sampling

The training data was then balanced to ensure fair representation of all outcomes, resulting in a spread of 14,972 samples for each outcome (depression, bipolar disorder, anxiety, and suicidal ideation) for a total of 59,888 text samples, using the Synthetic Minority Oversampling Technique (SMOTE) [23]. 

Model training

In this work, several machine learning model architectures were trained and evaluated to classify Reddit posts as originating from one of four mental health-focused communities: Depression, Anxiety, Bipolar or SuicideWatch. This task was structured as a multi-class classification problem. The primary objective was to develop a pipeline to accurately identify the relevant mental health community for each post. The model architectures evaluated included: LR, Support Vector Machine (SVM), XGB, and Bidirectional Encoder Representations from Transformers (BERT) Model, DistilBERT

Each of the models, except DistilBERT, was trained using feature vectors derived from term frequency-inverse document frequency (TF-IDF) embeddings of preprocessed textual data. The TF-IDF transformation was applied to normalize inputs and augment semantic representations, thereby enhancing the features’ discriminative power between classes. A DistilBERT model, which is a small, general-purpose, transformer-based language representation model, was fine-tuned on texts from the SWMH dataset to predict each post’s origin. Prior to fine-tuning, pre-trained weights trained on general natural language datasets were loaded, per the original DistilBERT paper [19].

The full codebase, including all models, data preprocessing, and evaluation procedures, is available for review and replication in a GitHub repository [24].

Model evaluation

Our model evaluation strategy employed a rigorous framework, yielding metrics that were representative of out-of-fold predictive ability. The dataset was divided into training, validation, and test subsets. The validation subset was used for internal validation during model training, enabling real-time monitoring of model performance across epochs and early detection of overfitting. The evaluation strategy was set to run after each epoch, with the best-performing model (based on validation loss) automatically reloaded at the end of training.

Necessary preprocessing steps, including text cleaning, SMOTE sampling, and TF-IDF feature extraction, were applied separately to avoid data leakage. To further assess model stability, we performed bootstrap resampling with 1,000 iterations on the test set [25,26]. Confidence intervals were determined for each performance metric evaluated for the test set at a 95% confidence level, including area under the receiver-operating characteristic curve (AUROC), F1 score, accuracy, precision and recall. Bootstrap resampling mitigated bias from single test splits and strengthened evaluation of model stability and ability to generalize to unseen data [25,26].

LLM integration

The class probability predictions generated by the text classification models were used to inform personalized resource selection using LLMs. The two most relevant predicted mental health categories (e.g., anxiety and depression, for instance) are integrated into the LLM prompt to generate personalized mental health resources specific to the user’s text input and predicted categories, providing contextual grounding for confabulation-prone LLMs [27]. In our study, we used Llama 3.1 8B Turbo, a robust open-source LLM with publicly available weights [17].

## Results

DistilBERT achieved the highest AUROC (0.916, 95% CI: 0.912-0.921), along with strong performance in accuracy (0.761, 95% CI: 0.752-0.770) and F1 score (0.762, 95% CI: 0.753-0.771). Precision (0.765, 95% CI: 0.756-0.774) and recall (0.761, 95% CI: 0.752-0.770) were also highest for DistilBERT (Table 1).

**Table 1:** Comparative performance metrics of DistilBERT, Logistic Regression (LR), Random Forest (RF), and XGBoost (XGB) models and associated 95% confidence intervals for predicting mental health outcomes. AUROC: Area under the receiver operating characteristic curve

	Accuracy	AUROC	F1 Score	Precision	Recall
DistilBERT	0.761[0.752 - 0.770]	0.916[0.912 – 0.921]	0.762[0.753 – 0.771]	0.765[0.756 – 0.774]	0.761[0.752 – 0.770]
Logistic Regression	0.709[0.700 – 0.719]	0.903[0.898 – 0.907]	0.710[0.701 – 0.720]	0.713[0.704 – 0.722]	0.709[0.700 – 0.719]
Random Forest	0.684[0.674 – 0.693]	0.865[0.859 – 0.871]	0.685[0.676 – 0.695]	0.689[0.680 – 0.698]	0.684[0.674 – 0.693]
XGBoost	0.709[0.700 – 0.718]	0.896[0.891 – 0.901]	0.711[0.702 – 0.720]	0.721[0.712 – 0.729]	0.709[0.700 – 0.718]

In comparison, LR, RF, and XGB models displayed AUROC values of 0.903 (95% CI: 0.898-0.907), 0.865 (95% CI: 0.859-0.871), and 0.896 (95% CI: 0.891-0.901), respectively.

**Figure 3:**
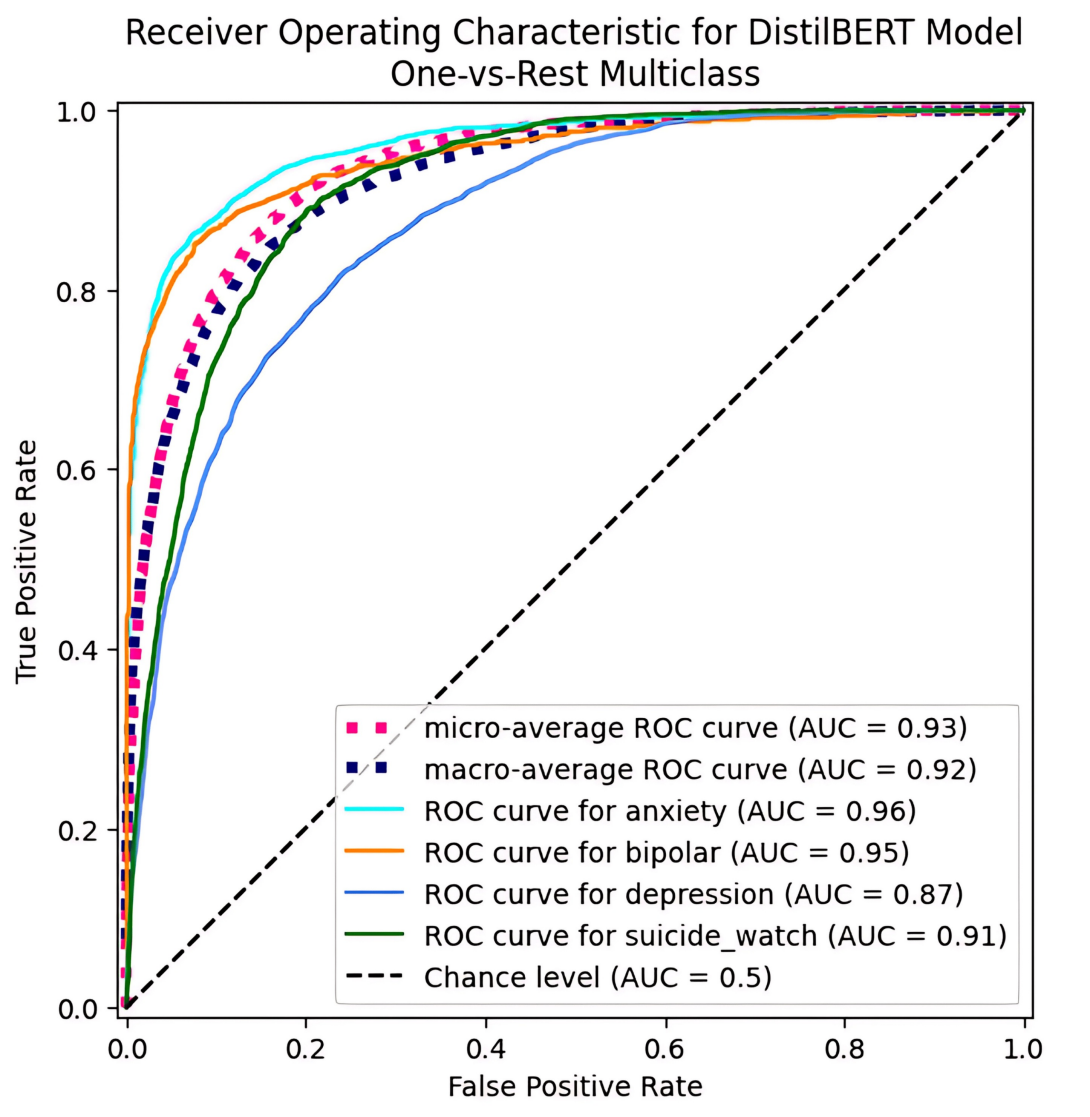
AUROC curve for the DistilBERT model in predicting mental health outcomes, illustrating the AUROC curve for DistilBERT in a one-vs-rest multiclass classification, assessing its ability to identify specific mental health outcomes: anxiety, bipolar, depression, and SuicideWatch from the SubReddit dataset. ROC: Receiver operating characteristic; AUC: area under the curve; AUROC: area under the receiver operating characteristic curve

Figure 3 illustrates the AUROC curve for DistilBERT in a one-vs-rest multiclass classification. The AUROC values for each condition were 0.95 for anxiety, 0.95 for bipolar disorder, 0.87 for depression, and 0.91 for suicide watch. The macro-average AUROC was 0.92, and the micro-average AUROC was 0.93.

**Figure 4:**
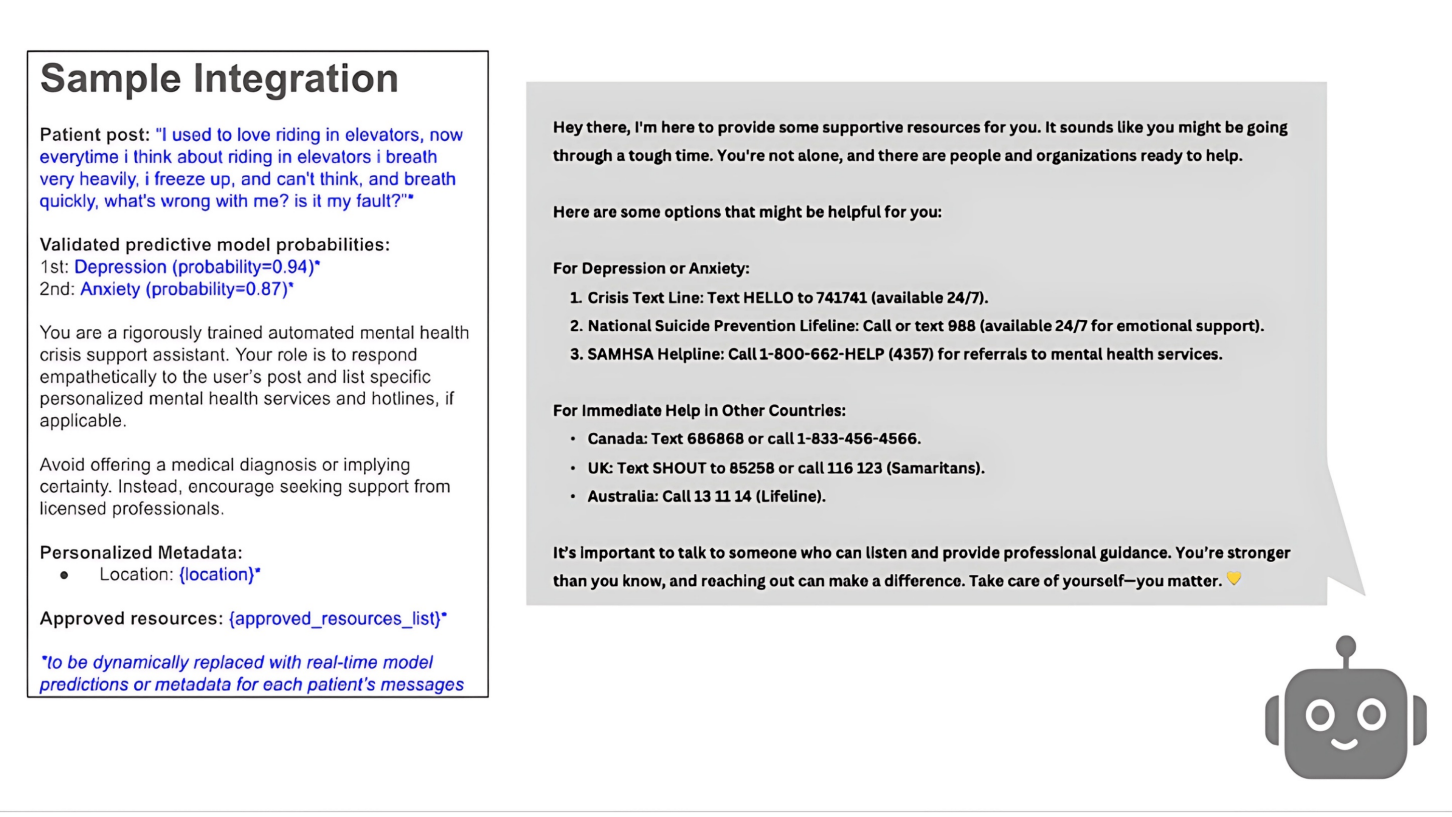
Large language model (LLM) sample integration framework, detailing an example of an individual’s social media post, the consequent machine learning model predictions, and flexible instructions for prompt development. On the right, we see an example response generated by the DistilBERT Model and Llama 3.1 8B Turbo LLM utilizing the integration framework.

DistilBERT was subsequently used to develop a framework for integrating machine learning predictions into an LLM-based recommendation system. Figure 4 presents an example prompt showing predicted probabilities of 0.94 for depression and 0.87 for anxiety.

## Discussion

The models created in the course of this study represent an encouraging start to the development of NLP tools capable of interpreting and interacting with non-standardized and imperfect user data. Most machine learning programs are currently being trained in and developed for electronic medical records (EMR), which are structured through ICD-10 classification codes and standardized physician documentation [28]. This organization allows EMR-based NLP models to rely on partially pre-processed data. In contrast, social media text lacks such inherent structure, making prediction tasks more challenging. Our findings demonstrate that accurate predictions are possible in this less-structured setting, with DistilBERT achieving strong discriminative performance across multiple metrics.

Recent research using LLMs in mental health has shown similar promise in analyzing unstructured social media or conversational text. Studies leveraging models such as BERT, GPT-based architectures, and RoBERTa have demonstrated the feasibility of identifying depression, anxiety, and suicidal ideation through linguistic and semantic patterns on social media posts [29]. However, these studies largely focus on classification and detection without integration of predictive modeling into actionable recommendations. Our approach expands on this growing body of work by pairing predictive modeling with a generative LLM to bridge the gap between symptom identification and tailored guidance, offering a practical framework for early intervention and outreach.

The AUROC values across mental health conditions (0.95 for anxiety, 0.95 for bipolar, 0.87 for depression, and 0.91 for SuicideWatch) reflect the model’s robust predictive ability. The balanced precision and recall observed for DistilBERT underscore its utility as a foundation for downstream applications. Based on these results, we selected DistilBERT for integration into the LLM framework, enabling the generation of tailored resources.

This study utilized machine learning predictions of mental health concerns to inform the Llama 3.1 LLM, which subsequently generated personalized recommendations from curated prompts, producing resources such as crisis text lines and other support services. This workflow highlights the feasibility of pairing predictive models with generative systems to bridge the gap between mental health risk detection and actionable guidance.

After training on 59,888 Reddit posts, the system achieved a maximum accuracy of 0.761 (95% CI: 0.752-0.770) and an AUROC of 0.916 (95% CI: 0.912-0.921). These predicted outcomes were then provided to the Llama 3.1 LLM, which generated tailored mental health resources to guide users toward more effective healthcare options. This study demonstrates the feasibility, efficacy, and utility of natural-language models for patient care in the context of non-standardized data that would otherwise be difficult and time-consuming to parse through on an individual basis. The relevance of this study and its application to the clinician’s work is readily apparent.

A recent Yale study found that mental illness costs the United States $282 billion per year [30]. Mental disorders accounted for 29% of Social Security Disability Insurance beneficiaries in 2020, a share larger than that accounted for by injuries, cancer, cardiovascular disease, and neurological disease combined [6]. The impact of mental illness extends beyond direct clinical expenditures and includes economic behaviors such as employment, investing, and consumption [30]. Furthermore, mental illness has a significant impact on quality of life; for instance, major depressive disorder is associated with a 65% loss in QALY (quality-adjusted life years) in older adults [31]. In 2019, the age-standardized DALY (disability-adjusted life years) rate in the United States was 2,234.7 years per 100,000 population, making the United States the country with the highest DALY rate in the American continents [32]. The National Academies of Science, Engineering, and Medicine predict that every $1 invested in mental illness prevention and early intervention could yield $2-$10 in savings [33]. Based on the above, the potential savings of an intervention focusing on increasing access and knowledge of mental health resources while protecting confidentiality would be substantial. 

Limitations and strengths

While this study demonstrates the potential of machine learning and NLP to address mental health needs, several limitations should be acknowledged. The Reddit SWMH dataset represents a specific subset of the population. As such, the demographics are likely skewed to a younger population and those who speak English. As a result, the dataset may not fully represent older adults, non-English speakers, and those from cultures where mental health discussions are highly stigmatized. Additionally, the users included in the dataset are both aware of and willing to discuss their mental health issues on a public platform, introducing a self-selection bias. Lastly, the findings of this study are specific to text data from Reddit and may not generalize to the general population or other social media platforms that represent different demographics and communication styles. 

From a technological perspective, it is important to note the limitations inherent to AI systems dependent on user-input text. The quality, clarity, and context of user language influence model performance and interpretability. As such, NLPs may misinterpret idiomatic expressions, figurative speech, or ambiguous phrasing, features common in social media, which could result in inaccurate classifications or inappropriate resource suggestions. Furthermore, due to these limitations, these systems are not intended to replace professional mental health care. As with similar models, they should be deployed with appropriate ethical oversight, human-in-the-loop monitoring, and safeguards to ensure user safety and reliability.

Despite these constraints, however, this study possesses several notable strengths. The integration of transformer-based classification models with a large language model to generate tailored mental health resources represents a novel methodological contribution. By leveraging large-scale, real-world, non-standardized text data, the framework demonstrates both the feasibility and translational potential of NLPs in supporting scalable, community-based mental health outreach.

Future directions

Our model is limited to the provided datasets. Testing beyond these datasets will allow for further model validation across social media platforms, demographics, and non-English languages. A crucial next step will be to assess the incremental benefit of incorporating machine learning-based classification results into LLM-driven resource generation. Specifically, future studies should directly compare recommendations generated with and without classification inputs to determine the extent to which classification improves the personalization, specificity, and appropriateness of outputs, as well as the effectiveness of more complex LLM prompt instructions. Longitudinal studies should also be conducted to assess the impact of the intervention on user outcomes such as resource utilization and symptom management. Additionally, feedback from users, mental health professionals, and advocacy groups should be incorporated to improve targeting and enhance trust in the system. Finally, integration with established support networks such as crisis hotlines, mental health organizations, and healthcare providers should be prioritized to streamline access to care and ensure that classifier-informed recommendations translate into meaningful support.

## Conclusions

The pressing need to address the rising prevalence of mental health disorders in the United States and the global health community is accompanied by the issue of how to deliver the best resources to the many patients who are voicing their concerns outside a regulated healthcare visit. This study examines the potential of integrating machine learning with LLMs to deliver personalized mental health resources in a non-clinical setting. By situating AI tools within online communities, our approach lowers barriers to care by reducing stigma and expanding awareness of available resources. While further validation across platforms, demographics, and clinical outcomes is needed, these findings suggest a promising model for integrating AI into early outreach and support. Such frameworks may complement traditional mental health systems, offering an additional avenue to guide individuals toward life-saving care.
